# A Holistic Approach of Personality Traits in Medical Students: An Integrative Review

**DOI:** 10.3390/ijerph182312822

**Published:** 2021-12-05

**Authors:** Nicoleta Suciu, Lorena Elena Meliț, Cristina Oana Mărginean

**Affiliations:** 1 European and Research Projects Department, “George Emil Palade” University of Medicine, Pharmacy, Sciences and Technology, 540136 Târgu Mureș, Romania; nico.suciu03@gmail.com; 2 Department of Pediatrics I, “George Emil Palade” University of Medicine, Pharmacy, Sciences and Technology, 540136 Târgu Mureș, Romania; marginean.oana@gmail.com

**Keywords:** personality, clinical communication, medical students

## Abstract

Personality is one of the most crucial aspects of human life, since it influences all human behaviours in both personal and social life, and might also trigger important conflicts with a person’s surroundings in the setting of incompatible traits and characteristics. It is true that ‘one must be born’ for a certain medical specialty, but several components of personality might be educated with proper training. Increased levels of Conscientiousness, Agreeableness, and Openness associated with lower levels of Neuroticism might represent the key combination for achieving professional satisfaction in the medical profession. Medical students should receive proper interprofessional education, since effective interprofessional relationships among healthcare providers definitely improve patients’ safety. Empathy contributes to effective patient–physician communication, improving patient trust, compliance, and satisfaction, being positively correlated with Openness, Agreeableness, Conscientiousness and Extraversion. Emotional intelligence—the capacity to respond to one’s own and others’ emotions—was proven to contribute, in a synergistic way with empathy, to increasing empathic ability. Clinical communication skills represent a key component in medical students in order to achieve the best patient care, and they are certainly related and/or influenced by empathy, interprofessional collaboration skills, emotional intelligence and, especially, personality traits. Taking into account the complex interactions mentioned above, the implementation of effective courses based on these concepts in medical students, intending to promote the development of clinical communication skills, represents a real emergency, since it might result in a reduction in medical errors and subsequent related deaths. A thorough understanding of students’ personality is mandatory before designing these courses in order to provide a training tailored to their personality styles.

## 1. Introduction

The ways people think, feel, and behave impact their daily social interactions and should be properly assessed and trained whenever possible, especially in terms of professions that require interhuman interactions. The medical profession is by far the most peculiar of all in terms of communication, since it might act dichotomously resulting in either a strong doctor–patient partnership or, on the contrary, in a poor relationship defined by a lack of trust and respect, which will have a major negative impact on a patient’s treatment outcome. However, the importance of medical communication is not always fully acknowledged and valued properly during medical education; once medical students graduate and begin to practice, they become aware of this crucial gap in their education. Moreover, this reflects on their medical actions and decreases their quality considerably. Medical communication should be based on empathy, defined as a complex social emotion consisting of the ability to identify and understand the feelings and thoughts of others and to respond with adequate emotions [1,2,3]. In terms of the medical profession, the concept of empathy was specified as being defined as a major cognitive attribute that implies the quality of understanding a patient’s concerns, experiences and perspective, as well as communicating this perception with the aim of helping them [4]. Thus, we emphasise the close interdependence between personality, medical empathy and communication. Medical education should consist of a holistic approach in terms of implementing and monitoring appropriate training strategies for developing proper communication skills, but also for shaping certain personality traits in order to achieve the best level of communication with the future patient. Nevertheless, it is a well-documented fact that personality traits are influenced mostly by genetic background [5], a fact that might represent a real challenge in medical undergraduates, taking into account that genetic inheritance is the most difficult to be modified or trained. Albeit minor, the influence of social skills on the development of personality should not be neglected, since it is the main component that could be modified with proper education.

Personality traits were also proven to be essential for medical students in choosing the most suitable specialty [6] based on a proper assessment and fair acknowledgement of their self-strengths and limitations. This takes into account that professional satisfaction influences not only the doctor’s quality of life and performance, but also the patient’s outcome. Thus, medical educators should also focus their attention on guiding undergraduates in making the best choice based on their personality and personal interests. Several studies proved that medical students reported personal satisfaction and interest as the most important factors that influenced their decision when choosing their specialty [7]. As additional proof of the complex relationship between personality traits, medical empathy and communication were stated by Hojat et al., who emphasised that a proper understanding of this relationship would not only guide specialty choice, but could also be helpful in selecting students that match the medical profession by predicting possible behaviours [4]. Medical performance represents the top of the pyramid built on personality traits, medical empathy, and communication—the final supreme goal of each physician.

Interprofessional collaboration, teamwork and multidisciplinary approach represent highly desirable skills in the medical profession, taking into account the current medical trends that support and focus on inter- and multidisciplinary medical teams for conveying patient safety, trust, and improving their outcomes [6]. The World Health Organisation emphasised the major importance of interprofessional teamwork in the medical field by implementing medical training focused on developing students’ collaboration skills [8]. However, certain personality traits such as neuroticism and introversion might display a negative influence on interprofessional collaboration if not shaped properly during medical school in order to diminish traits that alter this collaboration as much as possible.

Based on these aforementioned facts, we might state that personality traits represent the core of medical performance, irrespective of their chosen specialty, and a thorough understanding and assessment of these traits are the missing puzzle piece for implementing proper medical training and improving patient outcomes.

Thus, the main aim of this narrative overview was to underline the impact of personality traits on a physician’s work, academic satisfaction, and their patient’s outcome.

## 2. Literature Search

**Figure 1:**
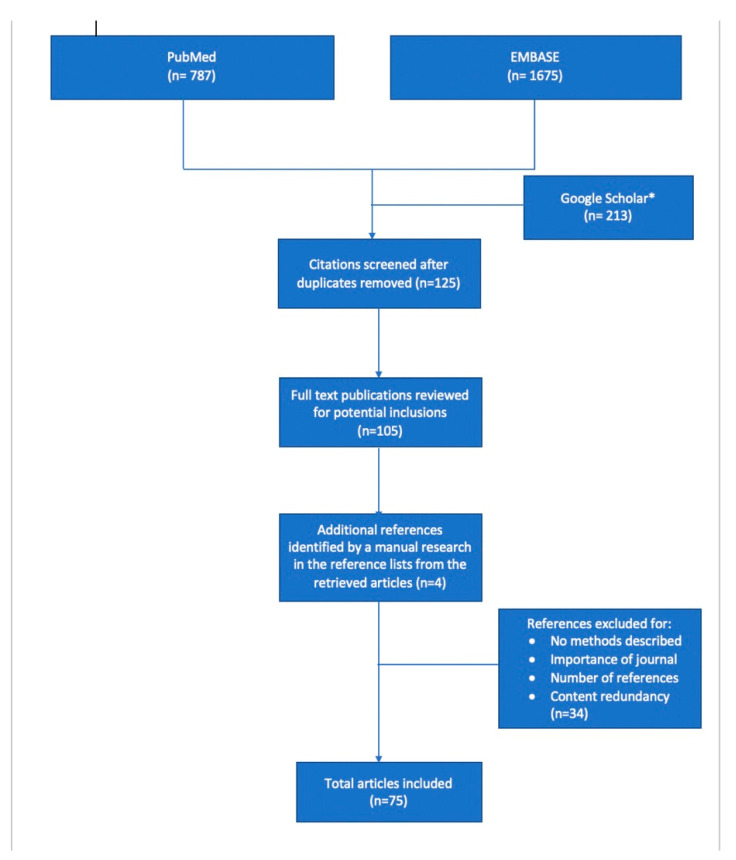
Flowchart of the literature selection. * Google Scholar was an auxiliary database used if the full text of a particular article was not found in PubMed or Embase.

An electronic literature search was performed for the present review on the lines of search for a narrative overview, including three databases: PubMed, Google Scholar and EMBASE. We used the following search terms: ‘personality traits’, ‘medical empathy’, ‘interprofessional collaboration’, ’career choice’, ‘emotional intelligence’, and ‘professional satisfaction’ (Figure 1). We should mention that Google Scholar was an auxiliary database used if the full text of a particular article was not found in PubMed or Embase. The inclusion criteria consisted of all types of articles that involved medical students, young residents or physicians, irrespective of the year of publication, in order to also highlight the progress of medical education in terms of acknowledging the importance of personality traits in the professional development of medical students. Each of the included articles was critically assessed according to the following: key results, quality of the results, interpretation of the results, suitability of the methods used to verify the hypothesis, limitations, and impact of conclusions to the field [9]. Moreover, we applied critical reading tools for scientific articles according to Jean-Baptist du Prel et al. [10], by analysing the study design of each article, the structure, the role of each section, as well as the potential sources of bias and limitations. Thus, analysis and inference were the most reliable critical reading tools used for the assessment of articles included in this review. We excluded articles that did not fit to the aforementioned criteria, as well as articles that were not written in English and those for which the full text was not available. We also identified additional references that fulfilled our criteria by a manual search of the reference list from the articles retrieved in the first round of search, for which we followed the same steps before including them in the review. We synthetized the findings from the remaining articles, highlighting both the similarities and inconsistencies between them in order to provide an objective narrative overview related to the impact of personality traits on a physician’s work, academic satisfaction, and their patient’s outcome. The methodological characteristics of the research-type studies included in this review are described in Table 1.

## 3. Personality Traits in Medical Students—The Core of the Doctor–Patient Relationship

As mentioned above, each individual has their own way of thinking, behaving and expressing feelings or emotions, characteristics that define an individual’s personality. Moreover, personality is known as one of the most crucial aspects of human life [11]. It is also known to influence all human behaviours in both personal and social life, but it might also trigger important conflicts with a person’s surroundings in the setting of incompatible traits and characteristics [12]. Two famous personality theorists, Zweig and Webster, emphasise that the differences in an individual’s personality represent probably the most important factor that influences a human’s motivation in learning, performance, and behaviour, considering that all the controversies in areas such as thinking, intelligence, perception, emotions, learning, and motivation are based on this issue [11]. Taking into account that effective doctor–patient communication represents the cornerstone of patient- and family-centred care, medical training should focus on improving personality traits that influence this communication. Nevertheless, this fact cannot be achieved without a proper assessment of personality traits.

One of the most frequently used tools for assessing personality is represented by the ‘big-five model’ and its derivatives, e.g., Personality Inventory NEO-PI-R [13]. The big-five model or the five-factor model has been used for assessing intercultural differences among different countries, defining the underlying qualities of personality traits [57,58]. Thus, the big-five model is based on the assessment of five dimensions of personality and its related facets, six for each dimension: Openness, facets—fantasy, aesthetics, feelings, actions, ideas, values; Extraversion, facets—warmth, gregariousness, assertiveness, activity, excitement-seeking, positive emotions; Conscientiousness, facets—competence, order, dutifulness, achievement striving, self-discipline, deliberation; Agreeability, facets—trust, straightforwardness, altruism, compliance, modesty, tender-mindedness; Neuroticism, facets—anxiety, angry hostility, depression, self-consciousness, impulsiveness, vulnerability [14,59]. Each of these dimensions is related to specific personality aspects and defines particular personality subdimensions. Thus, Openness is related to an individual’s intellectual interests, culture and openness to experiences [15]. Extraversion is a reliable indicator of energy, enthusiasm, sociability, orientation towards others and task perseverance [60,61]. Conscientiousness is another personality dimension that implies prudence, responsibility, moral integrity, the need for structure and order, as well as perseverance in action [60]. Interpersonal relationships represent a fair reflection of agreeability, which is related to altruism, goodwill, gentleness, direct behaviour and trust in people [60]. Emotional Stability or Neuroticism is associated with depression, impulsivity, anxiety, vulnerability and the tendency to worry [60]. This model was assessed in multiple general studies, but unfortunately, its utility in medical students is far from being properly assessed. Taking into account the great impact of their personality on their professional skills and performance, a proper understanding of their personality would be of great benefit during medical school.

Professional satisfaction is a strong hallmark of wellbeing during adulthood. In terms of the medical profession, this is choosing a specialty that matches best with an individual’s personality traits. Multiple factors were identified as essential contributors for medical specialty choice such as personality, gender, personal interest, economic status, clinical experience during clerkship, mentoring from professor, expected income, lifestyle, family or public media influence [16,17,18]. Thus, extrinsic factors display a greater influence on a student’s decision when choosing their specialty. Nevertheless, personality remains a major determinant in specialty decision making. Taking into account the major impact of this choice on public medical services and policy on medical education, each career counsellor or professor that mentors medical students should focus more on finding an effective method to assess their personality [62]. Moreover, a proper assessment of personality traits might predict performance in carrying out tasks entailed by each medical specialty [6]. Thus, we are definitely entitled to say that ‘one must be born’ for a certain medical profession in order to achieve the desired performance. A recent study performed on medical students aimed to identify the relationship between specialty choice and personality traits, and concluded that students with more Agreeableness preferred clinical medicine instead of basic medicine [6]. The authors also underlined that students with an increased level of Openness intended to choose medical departments [6]. Openness was also proved to be directly related to satisfaction and personal interest when choosing the medical specialty [6]. Unsurprisingly, age was shown to influence certain personality traits such as Conscientiousness and Agreeableness, which seem to be higher in older students [6]. Another study that compared surgical residents with the general population in terms of personality traits pointed out that surgeons scored significantly higher for Extraversion, Conscientiousness and Openness [19]. According to the study of Hoffman et al., who compared the personalities of surgical residents, nonsurgical residents and medical students, surgical residents scored better for Conscientiousness and Extraversion, but worse for Openness than medical students [20]. Thus, previous studies emphasised the fact that surgeons possess a particular personality distinct from that of trainees in other medical specialties or the general population [19,63], suggesting there is an innate character of their personality that enables them to perform this specialty. Nevertheless, the question of whether surgeons are born or made is rather challenging. Thus, a recent study performed on surgical trainees and medical students who intend to pursue a surgical specialty proved that the two groups display multiple similarities regarding both personality traits and learning styles [21], indicating that most likely an innate personality is required for this profession and these traits are neither learnt or developed during surgical training. Both groups included in this study scored high for Conscientiousness, Agreeableness and Openness, and low for Neuroticism [21]. These findings suggest that trainees adjust their Neuroticism during residency programs and become more emotionally stable over the course of surgical training, experiencing lower anxiety and depression levels as compared to undergraduates that aim to pursue a surgical career. Moreover, the combination between increased levels of Conscientiousness, Agreeableness and Openness and lower levels of Neuroticism might represent the key attribute for enabling students to accept better and cope with the tasks and rigor of a stressful residency program such as surgical training. Thus, personality testing should be mandatory during medical education since it was clearly showed that personality profiling is an extremely useful tool for distinguishing between high- and low-performing residents [22]. Taking into account the crucial impact of the match between personality traits and medical career on the health care services and national economy, each university should implement a program designed for personality testing during the medical training in order to properly guide medical students in their specialty choice.

Interprofessional collaboration between healthcare providers is essential in order to achieve the best outcome for each patient and improve patient safety. Thus, the collaboration of different healthcare professionals in well-functioning teams by contributing with their specific knowledge related to each medical specialty, resulting in a holistic approach, will definitely impact both medical performance and patient satisfaction [22,64]. Interprofessional education is meant to provide students opportunities to develop, train, and learn collaboration skills and each medical university should implement this type of education as a regular training, since it is often challenging to achieve good interprofessional teamwork [64]. It is true that awareness regarding the need for interprofessional education during medical school has increased considerably during the last few years, but students’ attitudes towards this type of training remain an issue since they seem to be influenced by multiple factors such as gender, learning styles or personality traits [8,23,24,25,26,27]. Thus, once more, we emphasise that a thorough understanding of personality traits in medical students could considerably improve their attitudes towards interprofessional education and collaboration by providing teachers the opportunity to design proper teaching methods adjusted to medical students’ personality traits and their preferences for different learning styles.

Medical empathy, defined as the ability to understand a patient’s experiences, concerns, and perspectives and to express this understanding in their favour [24], is another essential concept that contributes to effective patient–physician communication, improving patient trust, compliance, and satisfaction in order to achieve the best outcome in clinical practice [28]. Thus, it was fairly stated that the physician’s performance, clinical skills, and biomedical knowledge are strongly influenced by empathy and emotional abilities, which, in turn, are shaped by personality [29]. In terms of personality traits, Openness and Agreeableness were proven to be positively correlated with empathy [30]. Moreover, it seems that empathy is also positively correlated with two other personality dimensions, i.e., Conscientiousness and Extraversion [1]. Nevertheless, it is also true that increased empathy might result in emotional fatigue, increased risk of stress, and subsequent ‘burn out’ [65]. Self-assessment and understanding of personality traits could considerably decrease the previously mentioned risks by approaching suitable training strategies in order to improve unfavourable traits. The main daily goal for each physician is to provide the best of care for their patients and, therefore, the relationship between empathy, emotional intelligence, and personality was found to be the main factor that contributes to successful achievement of this goal. Multiple studies proved that emotional intelligence is associated with increased empathy, improved patient–physician relationship, teamwork, communication skills, stress management, and leadership resulting in higher academic performance [31,32,65]. The relationship between emotional intelligence and personality is further emphasised by the fact that the first one includes the ability to understand, perceive, and manage self-emotions, but also to perceive and understand the emotions of other humans [66]. In spite of their inherited and innate pattern, studies proved that emotional intelligence and empathy can be improved through proper education, implying an urgent need to implement communication training in medical students, since they seem to have a positive influence on both emotional intelligence and empathy [33,34,67]. Thus, an effective program designed for enhancing emotional intelligence and empathy in medical students should definitely take into account students’ personality [35,67,68]. The implementation of such a program during medical school is crucial, since it was proved that empathy declines during academic medical school training [36]. In addition, the decreasing pattern of empathy continues after they begin to practice their profession, since a doctor’s life is best characterised by resulting in an impairment of personality traits and devotion to patients. Therefore, programs for enhancing empathy would be of major benefit for improving professional experiences and performance.

A close interplay between personality traits, empathy, and interprofessional collaboration was emphasised by recent studies, which proved that empathy is positively associated with collaborative interprofessional work in postgraduates who are at the beginning of their specialisation [37], being well-documented that both empathy and interprofessional work are influenced by personality traits. Moreover, it was underlined that general empathy might be predicted by different personality styles, such as styles of protection, sensation, and accommodation [38]. In terms of interprofessional collaboration and communication skills, it was noticed that individuals that display a greater degree of empathy find it easier to accept failures in their life, being more optimistic regarding the future, motivated to satisfy others first, and trying to change their environment to fulfil their goals [38]. According to Zaki et al., empathy is a motivated phenomenon implying a close interaction between social desirability and intrinsic motivation [69]. Thus, a student’s self-efficacy is moderated mainly by two motivational factors, i.e., value of the goal for which they work and the expectation to achieve that goal [39]. Nevertheless, intrinsic motivation and self-efficacy are not enough to achieve the goal in medicine if not supported by empathy. The complexity of practicing and understanding empathy in clinical situations requires a solid basis provided by intense training in terms of personal development, relationship and system maintenance, chance, and goal progress [40].

Emotional intelligence is another crucial ability for medical students, since it represents a measure of emotional awareness and the capacity to respond to one’s own and others’ emotions. Thus, Mayer and Salovey stated that emotional intelligence ability implies not only the ability to perceive and integrate emotion, but also to understand and regulate emotion in order to favour personal growth [29,70]. The Accreditation Council for Graduate Medical Education defined emotional intelligence competency as a set of assessment methods for interpersonal and communication skills, patient care, and professional behaviours in residents [71]. Thus, 12 abilities were related to emotional intelligence competency: emotional self-awareness, emotional self-control, achievement orientation, adaptability, empathy, positive outlook, coach and mentor, organisational awareness, influence, inspirational leadership, teamwork, and conflict management, which represent, along with empathy, the most important traits of 21^st^ century physicians [41]. An increased level of emotional intelligence was shown to positively influence doctor–patient relationship, teamwork, communication skills, level of empathy, organisation commitment, as well as stress management [31,32,65,72]. Emotional intelligence and empathy synergistically contribute to increasing empathic ability [29]. One of the most important deficiencies in medical students is a lack of confidence and resources to implement empathic behaviour, despite them fully acknowledging and understanding its value [29], emphasising once more the need for proper dedicated training during medical school. Therefore, medical education should focus not only on improving empathy and empathic behaviour in medical students, but also promoting and teaching methods for its effective implementation in residents and young physicians. Emotional intelligence is definitely influenced by personality. A recent study performed on US medical students, which focused on assessing emotional intelligence, revealed a strong positive association with Extraversion, a moderate positive one with Conscientiousness, Agreeability and Openness, as well as a weak negative association with Neuroticism [35]. Moreover, Conscientiousness and Openness were found to be reliable predictors for medical students’ competencies in clinical contexts [42]; Openness and Agreeability promote the development of patient–physician relationships [43], as well as training proficiency [73]; and Conscientiousness was identified as the most important trait required for effective task completion [74]. Considering the complex aforementioned findings, emotional intelligence has an incontestable influence on patient–physician relationships, medical students’ and physicians’ communication skills, professional performance, and proficiency, creating important bridges with empathy and interprofessional collaboration skills under the close guidance of personality traits.

Clinical communication skills represent the key component in medical students in order to achieve the best patient care, and they are certainly related and/or influenced by empathy, interprofessional collaboration skills, emotional intelligence, and especially personality traits. Nevertheless, effective communication between physician and patient/caregivers represents the ‘cornerstone’ of care by improving the patient’s outcome. Good communication skills improve not only the physical but also the mental health of patients, increasing their compliance and satisfaction regarding the provided care [44]. The physician’s ability to communicate and their knowledge represent two major factors that equally contribute to the patient’s outcome [45]. In addition, personality traits were proven to also affect attitudes regarding communication skills training, although these skills are currently considered a crucial attribute for all people working in primary health care systems [46]. Medical errors, a continuous cause of death displaying an increasing pattern worldwide, seem to be mainly related to a major lack of communication skills [47]. It is indeed a real fact that the current trends in medicine focus on the acquisition of proper communication skills in medical students and not only on enhancing the theoretical aspects of each medical field, underlining that these skills should be considered when determining physicians’ competence [11]. Thus, medical educators should pay close attention and combine students’ personality and the ability to learn the communication skills in order to develop effective communication skills training to reduce medical errors in the long run [45]. Based on these findings, we hypothesise that physicians’ appropriate and effective ability to communicate might result in a considerable reduction in medical errors and subsequent deaths. Medical educators should also focus on increasing students’ awareness regarding the importance of learning communication skills, since it was underlined that they find no merit in improving these skills because they do not acknowledge them as an important aspect of medical and practical education [11]. This fact is rather concerning considering the effectiveness of students’ attitudes and satisfaction with learning in motivating and promoting knowledge quality [45]. Recent studies focusing on medical students’ personality traits and their attitude towards learning communication skills concluded that anxiety was associated with a negative perspective regarding communication skills training during medical school [48,49], while students with increased sociability levels were found to have a positive attitude towards this training [45,48]. Moreover, Agreeableness, Extraversion, and Conscientiousness were positively correlated with medical students’ attitudes regarding the interaction with patients, underlining that these personality dimensions might also be related to students’ attitudes in terms of communication [50]. Contrariwise, aggression/hostility represents the main characteristic of antisocial and bold behaviours, and it was associated with a negative attitude towards learning communication skills, since individuals with this type of personality express no interest in communicating with others by lacking this desired attitude [45].

The importance of clinically focused skills and behaviours in medical education has been acknowledged by both the Association of American Medical Colleges and the European Board of Internal Medicine for over a decade [75,76]. The competencies related to clinical communication skills and to clinical practice include trust building; respect; compassion; altruism; empathy; understanding patient’s spirituality, beliefs and meaning; professionalism; information sharing and mutual decision making; relationship building; cultural sensitivity; responsiveness to distress; appreciation of psychological factors; self-awareness; ethical issues; and acting with honesty [75,76]. These competencies were related to personality dimensions in several studies, but the results remain contradictory depending on the studied populations. Therefore, Sims et al. assessed the relationship between personality traits and communication skills in adults and showed that Agreeableness and Openness might predict assertiveness and empathic listening in communication [51]. Contrariwise, a study performed on psychology students revealed no significant correlation between the same neither before nor after communication training [52]. Studies performed on medical students remain scarce. Nevertheless, Agreeableness, Openness, and Extraversion were found to be the most important factors that positively influence students’ attitude towards communication and learning, enhancing a better connection between the patient and physician [53]. Moreover, each of these dimensions were proven to have a major contribution to achieving the ultimate goal of improving students’ attitudes towards communication in different ways: Agreeableness enables students to initiate a relationship with patients easier by providing the tendency to be friendly [54], Openness promotes the better acceptance of adversity and willingness to change [55], while Extraversion favours energy to social interaction better expressing the inner disposition [56]. Based on all these evidence-based statements, medical teachers should focus more on assessing students’ personality traits and implement their teaching activities tailored accordingly or even support the development of personality dimensions that favour an effective doctor–patient communication.

## 4. Conclusions

The relationship between physician and patient represents the core of medicine and all efforts should focus on improving this relationship. A thorough assessment of medical students’ personality traits is crucial for educators in order to acknowledge the main deficiencies in their students and to design effective tailored communication and clinical practice training for improving and educating these deficiencies. Professional satisfaction is definitely influenced by career choice, which should be properly guided after an individual assessment of personality traits. Interprofessional collaboration and teamwork are mandatory for patient safety and medical students should be made aware of its importance even before graduating. Emotional intelligence and empathy act as partners for improving empathic ability in order to reach the best doctor–patient relationship. Empathy is positively influenced by being positively correlated with Openness, Agreeableness, Conscientiousness, and Extraversion, and thus, training should focus on improving these components in medical students. The ultimate goal of the medical profession is to achieve the best outcome for each patient. Empathy, emotional intelligence, and clinical communication skills are essential for building an effective doctor–patient relationship, while interprofessional collaboration and teamwork are mandatory for assuring the best of care in practice, altogether being closely governed by personality traits and crucial for providing the best outcome for the patients. Taking into account students’ personality traits, courses should aim to improve empathy, clinical communication skills, interprofessional collaboration, teamwork, and practical competencies for the patients’ best outcomes. Thus, proper training should begin in medical students and should never end.

## Figures and Tables

**Table 1:** The methodological characteristics of the research-type studies included in this review.

Author, Year, and Country	Title of the Study	Aim	Study Type	Sample	Median Age or Mean (Years)/Gender (%)	Methods/Scales	Observations
Guilera et al., 2019, Spain [1]	Empathy and big five personality model in medical students and its relationship to gender and specialty preference: a cross-sectional study.	To explore the relation-ship between *empathy* and personality	Prospective cross-sectional study)	110 medical students	22/76.4% F	JSPE IRI EQ NEO-FFI	Empathy is related to personality
Baron-Cohen et al., 2004, United Kingdom [2]	The empathy quotient: an investigation of adults with Asperger syndrome or high functioning autism, and normal sex differences	To test EQ score in high-functioning autism (HFA) and Asperger Syndrome (AS)	Prospective case–control study	180 high-functioning autism (HFA) and Asperger Syndrome (AS) subjects	34.2/72.22% M	EQ WAIS-R	Empathy deficit in AS/HFA
Hojat et al., 2015, USA [4]	Eleven years of data on the Jefferson scale of empathy-medical student version (JSE-S): proxy norm data and tentative cutoff scores	To obtain cutoff scores for the JSE S-version	Prospective observational study	2637 medical students	23.4 ± 2.4 SD/50.66% F	JSE-S	Usefulness of empathy in decision making
Yusoff et al., 2018, Malaysia [5]	Which personality traits have favourable impact on psychological health during stressful condition?	To investigate which personality traits have a favourable impact on the psychological health of medical students during the most stressful period	Prospective cross-sectional study	174 medical students	19.27/67.2% F	Personality type DASS-21 USMaP-i	NEO-FFI Favourable impact on neuroticism, unfavourable impact on stress
Kwon et al., 2016, Korea [6]	Specialty Choice Preference of Medical Students According to Personality Traits by Five-Factor Model.	To evaluate personality by NEO-FFI	Prospective cross-sectional study	110 medical students	28.9 ± 2.1 SD/ 39% F	Personality traits by NEO-FFI	Personality important in choosing medical specialty
McCrae et al., 1989, USA [7]	Reinterpreting the Myers-Briggs Type Indicator from the Perspective of the Five-Factor Model of Personality.	The MBTI indicator was evaluated from the perspectives of Jung’s theory of psychological types and the five-factor model of personality on the NEO-PI	Prospective observational longitudinal study	468 adults	62.7 M and 58.9 F/57.05% M	MBTI indicator NEO-PI Psychological types	To reinterpret the MBTI in terms of the five-factor model
Olsson et al., 2020, Sweden [8]	Personality and Learning Styles in Relation to Attitudes towards Interprofessional Education: A Cross-Sectional Study on Undergraduate Medical Students during Their Clinical Courses	To investigate the effect of personality traits and learning styles on medical students’ attitudes towards IPE	Prospective cross-sectional study	79 medical students	29/63% F	NEO-FFI IPE	No correlation between personality, learning style and attitude towards IPE
Tamannaeifar et al., 2014, Iran [11]	The relationship between personality characteristics, social support and life satisfaction with university students’ academic performance	To investigate the relationship among personality characteristics, social support and life satisfaction with academic performance	Prospective descriptive, correlative survey	250 humanities’ students	-/-	NEO-FFI MOS-SSS SWLS	Relationship between neuroticism and conscientiousness with academic performance
Nasri et al., 2017, Iran [12]	Personality characteristics, irrational beliefs, and communication skills as predictors school counselors’ job performance.	To predict counsellors’ job performance based on their personality	Prospective clustering random sampling method	283 counsellors working	-/-	NEO-FFI	Personality characteristics are predictors of counsellors’ job performance
Vassend et al., 2011, USA [13]	The NEO personality inventory revised (NEO-PI-R): Exploring the measurement structure and variants of the five-factor model	To evaluate NEO-PI-R personality	Prospective study	856 adults	50.8/55.84% F	NEO-PI-R	NEO-PI-R reflects personality
Lievens et al., 2002, Belgium [14]	Medical students’ personality characteristics and academic performance: a five-factor model perspective	To evaluate NEO-PI-R personality	Prospective cross-sectional inventory study and prospective longitudinal study of one cohort of medical students	785 Flemish medical students and 1361 students from Ghent University	18.2/60% F	NEO-PI-R	Personality assessment—a useful tool in student counselling and guidance
Han et a., 2017, USA [15]	Big five personality factors and facets as predictors of openness to diversity	To examine the associations between NEO-FFI higher order factors and lower order factors and universal-diverse orientation	Prospective cross-sectional study	Study 1—388 medical students and Study 2—176 undergraduates	21.18/58.6% F—study 1 21.01/72.2% F—study 2	NEO-FFI UDO	Practical implications on how personality factors are incorporated into current diversity interventions
Borges et al., 2008, USA [16]	Emotional intelligence and medical specialty choice: findings from three empirical studies	To examine emotional intelligence (EI) and specialty choice among students at three US medical schools	Prospective observational study	84 medical students—study 1 250 medical students—study 2 292 medical students—study 3	24.3/50% F—study 1 22.7/44.8% F—study 2 23.6/38% F—study 2	MSCEIT ^TM^ study 1 TMMS and IRI, study 2 EQ-I^®^, study 3	No significant differences in EI between students entering primary care and non-primary care specialties
Hojat et al., 2008, USA [17]	Personality and specialty interest in medical students	To evaluate the impact of personality score on career interests	Prospective observational study	1076 medical students	-/64% M	ZKPQ-S	Personalities of medical students predict their career interests
Lambert et al., 2005, USA [18]	The relationship between specialty choice and gender of U.S. Medical Students, 1990–2003	To assess the relationship between specialty choice and gender	Prospective observational study on 14 years	184262 medical school graduates	-/34.6% F	AAMC GQ	Women were not more responsible than men for from uncontrollable lifestyle specialties
McGreevy et al., 2002, USA [19]	Preliminary measurement of the surgical personality.	To test for a distinct surgical personality	Prospective observational study	39 surgical residents	/61.53% M	NEO-PI-R	Similarity of trait variance from the general population in both male and female surgical residents
Hoffman et al., 2010, United Kingdom [20]	Personality differences between surgery residents, nonsurgery residents, and medical students	To examine specialty group differences in personality traits	Prospective observational study	274 surgical residents and 207 medical students	/53% M	OCEAN personality	Greater levels of conscientiousness were observed in surgery residents
Preece et al., 2016, United Kingdom [21]	Are surgeons born or made? A comparison of personality traits and learning styles between surgical trainees and medical students	To score 5 personality domains (extraversion, conscientiousness, agreeableness, openness to experience, and neuroticism)	Prospective cross-sectional study	53 medical students and 37 surgical trainees	-/-	FFM	Similarities in the personality traits and learning styles of surgical trainees and students interested in surgical career
Foster et al., 2010, USA [22]	A psychological profile of surgeons and surgical residents	To determine work-related personality and interest variables	Prospective observational study	63 surgical residents and 27 attending/teaching surgeons	31.2/74.60% M surgical residents 49.3/ 92.59% M teaching surgeons	CIA JSI	WOWI Online assessment tool provides a stable profile of successful surgeons
Hayashi et al., 2012, Japan [23]	Changes in attitudes toward interprofessional health care teams and education in the first- and third-year undergraduate students	To assess the implementation of a lecture style for 1st year students and a training style for 3rd year students	Prospective observational study	285 medical students	-/-	ATHCTS RIPLS	Scores improved after the training-style learning approach was implemented in the third-year students
Wilhelmsson et al., 2011, Sweden [24]	Are female students in general and nursing students more ready for teamwork and interprofessional collaboration in healthcare?	To investigate if student characteristics have an impact on their open-mindedness about cooperation with other professionals	Prospective cross-sectional study	670 medical students	-/73.1% F	RIPLS	Indicates some directions for more successful interprofessional education
Alghasham et al., 2021, Saudi Arabia [25]	Effect of students’ learning styles on classroom performance in problem-based learning	To identify learning styles	Prospective observational study	65 medical students	-/73.84% M	Learning Style Inventory Questionnaire	Students should be informed about their preferred learning style
Tariq et al., 2016, Pakistan [26]	Association between academic learning strategies and annual examination results among medical students of King Edward Medical University	To find an association between academic learning strategies and annual examination	Prospective simple random sampling	300 medical students	-/56% F	Biodata pro forma ILS questionnaire	Females outperform their male counterparts in academic performance
Avrech Bar et al., 2018, Israel [27]	The role of personal resilience and personality traits of healthcare students on their attitudes towards interprofessional collaboration.	To examine the attitudes of nursing, occupational therapy and physical therapy students towards interprofessional collaboration	Prospective descriptive cross-sectional study	184 healthcare students	25.4 ± 2.8/16.8% M	IEPS CD-RISC BFI	IPE, including PBL, should be integrated in health profession students’ training
Hojat et al., 2011, USA [28]	Empathic and sympathetic orientations toward patient care: conceptualization, measurement, and psychometrics.	To develop instruments for measuring empathic and sympathetic orientations in patient care and to provide evidence in support of their psychometrics	Prospective observational study	201 medical students	-/-	JSE IRI Empathic and Sympathetic Care	The validated measures of empathic and sympathetic orientation provide research opportunities
Abe et al., 2018, Japan [29]	Associations between emotional intelligence, empathy and personality in Japanese Medical Students.	To investigate: (1) The association between empathy, EI, and personality (2) Gender differences in the association between empathy, EI, and personality	Prospective observational study	351 medical students	20.42/70% M	TEIQue-SF JSPE NEO-FFI	Medical students’ EI may be enhanced with thoughtful training
Costa et al., 2014, Portugal [30]	Associations between medical student empathy and personality: a multi-institutional study.	To assess associations across institutions, looking for personality differences between students with high empathy and low empathy levels	Prospective observational study	472 medical students	21/66.10% F	NEO-FFI JSPE	Medical schools may need to pay attention to the personality of medical students
Chew et al., 2013, Malaysia [31]	Emotional intelligence and academic performance in first and final year medical students: a cross-sectional study	To examine the effect of EI on academic performance in first- and final-year medical students	Prospective cross-sectional study	163 medical students	21.8 ± 1.98/68.7% F	MSCEIT	Emotional skill development may enhance medical students’ academic performance
Aithal et al., 2016, India [32]	A survey-based study of emotional intelligence as it relates to gender and academic performance of medical students	To assess trait EI, to examine possible differences in EI level, and to establish a correlation between the EI of medical students and their academic performance	Prospective cross-sectional survey	200 undergraduate medical students	-/107 F/95 M	TEIQue-SF	Positive correlation between EI and academic performance
Magalhães et al., 2012, Portugal [33]	Empathy of medical students and personality: evidence from the five-factor model	To test hypothetical associations between personality dimensions and empathy scores in medical students	Prospective observational study	350 medical students	-/69.8% F	NEO-FFI JSPE-spv	Personality of students should be taken into account in programs to enhance empathy
Hojat et al., 2013, USA [34]	Enhancing and sustaining empathy in medical students	To test the hypotheses that medical students’ empathy can be enhanced and sustained by targeted activities	Prospective control-group study	248 medical students	-/51% F	JSE	Medical students’ empathy can be enhanced and sustained
Bertram et al., 2016, USA [35]	Strong correlations between empathy, emotional intelligence, and personality traits among podiatric medical students: a cross-sectional study	To evaluate empathy levels in podiatric medical students in a 4-year doctoral program	Prospective cross-sectional observational study	150 medical students	-/53.3% F	EI JSPE NEO-FFI	Strong correlation between empathy, EI, and personality in podiatric medical students
Youssef et al., 2014, Caribbean [36]	An exploration of changes in cognitive and emotional empathy among medical students in the Caribbean	To explore the empathy profile of students across five years of medical training	Prospective comparative cross-sectional design	669 medical students	22–27 years/65% F	JSPE RMET TEQ	Medical students’ lower empathy scores appear to be due to a change in the affective component of empathy
San-Martín et al., 2016, Spain [37]	Empathy, inter-professional collaboration, and lifelong medical learning in Spanish and Latin-American physicians-in-training who start their postgraduate training in hospitals in Spain. Preliminary outcomes	To identify similarities and differences in empathy, abilities toward interprofessional collaboration, and lifelong medical learning, between Spanish and Latin-American physicians	Prospective observational study	156 physicians-in-training	24–50 years/63.46% F	JSE Jefferson scale attitudes	Positive influence of empathy in the development of interprofessional collaboration abilities
Dávila-Pontón et al., 2020, Ecuador [38]	Empathy and personality styles in medical students	To establish the relationship between empathy and personality styles in medical students	Prospective non-experimental, descriptive, cross-sectional study	278 medical students	20.88 ± 2.78/59.7% F	JSE MIPS	Female students present an average score of total empathy greater than men
Ratelle et al., 2007, Canada [39]	Autonomous, controlled, and amotivated types of academic motivation: a person-oriented analysis.	To investigate students’ profiles regarding autonomous, controlled, and amotivated regulation, and test whether profile groups differed on some academic adjustment outcomes	Prospective control group study	4498 high school students	14.97/50.28% M	Academic motivational profiles	Underscores the importance of studying students’ motivation using a person-oriented approach
Doménech-Betoret et al., 2017, Spain [40]	Self-Efficacy, satisfaction, and academic achievement: the mediator role of students’ expectancy-value beliefs.	To examine and identify underlying motivational processes through which students’ academic self-efficacy affects student achievement and satisfaction	Prospective observational study	797 secondary education students	12–17 years/ 50.7% M	Self-efficacy The achievement/satisfaction relationship	Students’ expectancy value beliefs played a mediator role between academic self-efficacy and the achievement/satisfaction relationship
Webb et al., 2010, USA [41]	Emotional Intelligence and the ACGME Competencies.	To evaluate the use of EI assessment and training tools in assessing and enhancing interpersonal and communication skills	Prospective control-group study	21 residents	-/-	ESCI	EI is a necessary skill in today’s health care
Gough et al., 1991, USA [42]	Performance of residents in anesthesiology as related to measures of personality and interests	To study personality variables	Prospective observational study	99 residents in anaesthesiology	-/-	CPI SII	Empathic sensing by the anaesthesiologist function positively
LePine et al., 2000, USA [43]	Adaptability to changing task contexts: effects of general cognitive ability, conscientiousness, and openness to experience	To examine the extent to which cognitive ability, conscientiousness, and openness to experience predict decision-making performance	Prospective observational study	73 undergraduates	-/-	BFIDecision making performance	Unexpected low conscientiousness made better decisions
Shankar et al., 2013, Caribbean [44]	Student attitude towards communication skills learning in a Caribbean Medical School.	to establish the attitude of students towards to communication skils	Prospective observational study	73 undergraduate medical students	20–25 years/47.1% M	CSAS	Students overall had a positive attitude towards communication skills
Akbarilakeh et al., 2020, Iran [45]	Predicating attitude toward learning communication skills in medical students of Shahid Beheshti University.	To investigate the attitude toward learning communication skills based on the personality traits of medical students	Prospective correlational study	234 medical students	-/65% F	CSAS Zuckerman–Kuhlman personality questionnaire contains 5 personality dimensions	The dimension of demographic characteristics are effective in improving the communication skills of medical students
Gheirati et al., 2016, Iran [46]	Relationship between communication skills and mental health among the students of Mashhad University Of Medical Sciences	To determine associations between communication skills and mental health	Prospective cross-sectional analytical study	210 medical students	-/-	Communication Skills Questionnaires General Health Questionnaires	To promote the mental health of the students, it is recommended to conduct psychological assessments of the students
Nami et al., 2014, Iran [47]	The Role of students personality traits on students learning style in university of medical sciences	To investigate the relationship between personality traits and Kolb learning style	Prospective observational study (MANOVA test)	300 medical students	-/-	Neo-FFI Kolb learning style questionnaire	Significant relationships among the components of personality traits and their learning style
Molinuevo et al., 2013, Spain [48]	Does personality predict medical students’ attitudes to learning communication skills?	To determine whether personality is related to medical students’ attitudes towards learning communication skills and self-ratings on communication skills	Prospective control-group study	1031 medical students (divided in 2 groups: 524 1st-year students and 507 2nd-year students)	18.89 ± 3.21 years—1st-year students/66% F 20.11 ± 3.10 years—2nd-year students/70% F	CSAS EPQ ZKPQ	Personality traits are useful for better student career guidance and counselling
Zare-Alamshiri et al., 2017, Iran [49]	Prediction of communication skills based on psycho-social class atmosphere and social anxiety of high school students	To predict the psychological atmosphere of social communication skills and social anxiety in high school students	Prospective observational study	210 high school students	-/-	Social anxiety scale CSAS classroom psychosocial climate scale	Psychosocial classroom atmosphere can predict communication skills and social anxiety
O’Tuathaigh et al., 2019, Ireland [50]	Medical students’ empathy and attitudes towards professionalism: relationship with personality, specialty preference and medical programme	To examine how empathy, personality, and background factors might impact students’ attitudes towards professionalism in medicine	Prospective cross-sectional questionnaire-based study	241 medical students	18–22 (46.6%), 23–27 (43.3%), 28–32 (8.8%), 33–37 (0.8%), 38–42 (0.5%)/49.2% F	JSE NEO-FFI Attitudes towards professionalism scale	Empathy and personality factors may act as determinants of students’ attitudes
Sims et al., 2017, United Kingdom [51]	Do the big-five personality traits predict empathic listening and assertive communication?	To investigate whether the Big Five had predictive influences on communication competences of active-empathic listening (AEL) and assertiveness	Prospective observational study	245 adults	<25 years 59; 26–35 years: 42; 36–45 years: 50; 46–55 years: 64; >56 years: 28/75.9% F	AEL IPIP RAS	Agreeableness and Openness uniquely predicted AEL Extraversion had the biggest influence on assertiveness
Kuntze etl., 2016, Holland [52]	Big five personality traits and assertiveness do not affect mastery of communication skills.	To investigate whether the big-five personality factors and assertiveness predict mastery of communication skills	Prospective observational study	143 bachelor students of a psychology curriculum	19/83% F	FFPI SIB CSPT	Trainees can become professional communicators, regardless of their scores on these personality factors
Franco et al., 2020, Brazilia [53]	The assessment of personality traits and its association with learning communication skills	To investigate the association between personality traits and attitudes toward learning communication skills in undergraduate medical students	Prospective observational study	204 students	-/-	CSAS BFMM	Elation between agreeableness, extraversion and openness to experience with attitudes on communication skills in students
Vermetten et al., 2001, The Netherlands [54]	The Role of personality traits and goal orientations in strategy use	To contribute to the development of an integrated theory on individual learning differences	Prospective observational study	310 students	21.5/75% F	ILS Goal orientation scale SITIQ Big Five personality factors	Individual differences in learning consist of and help explain regularities in learning behaviour
Holen et al., 2015, Norway [55]	Medical students’ preferences for problem-based learning in relation to culture and personality: a multicultural study	To explore positive and negative preferences towards PBL in relation to personality traits and sociocultural context	Prospective cross-sectional survey	449 medical students (123 from Nepal, 229 from Norway and 97 from the USA)	21.4 Nepal/34.9% F 22.1 Norway/55.28% F 24.1 USA/ 46.10% F	PBL	Preferences related to PBL were significantly and independently determined by personality traits and culture
Tsou et al., 2013, Taiwan [56]	Using personal qualities assessment to measure the moral orientation and personal qualities of medical students in a non-western culture	To select candidates with appropriate personal qualities for medical school	Prospective observational study	746 medical students	20.3 ± 2.2/ 65.8% M	Mojac and NACE scale of PQA Big Five” personality traits	Significant relationships were observed between test components and between the NACE and Big 5

**Legend:** AAMC GQ—Association of American Medical Colleges’ (AAMC) Medical School Graduation Questionnaire (GQ); AEL—active-empathic listening; AS—Asperger Syndrome; ATHCTS—modified Attitudes Toward Health Care Teams Scale; BFI—Big Five Inventory; BFMM—the Big Five Mini-Markers; CD-RISC—Connor–Davidson Resilience Scale; CIA—Career Interest Activities; CPI—California Psychological Inventory; CSAS—Communication Skills Attitude Scale; CSPT—Communication Skills Progress Test; DASS-21—21-item Depression Anxiety Stress Scale; EI—emotional intelligence; EPQ—Eysenck Personality Questionnaire; EQ—the Empathy Quotient; EQ-I^®^—Bar-On Emotional Quotient Inventory; ESCI—Emotional and Social Competence Inventory; FFM—International Personality Item Pool Big-Five Factor Marker; FFPI—Five Factor Personality Inventory; HFA—high-functioning autism; F—female; ILS—Index of Learning Style; IPE—interprofessional education; IEPS—The Interdisciplinary Education Perception Scale; IPIP—Big-Five factor markers from the International Personality Item Pool; IRI—Interpersonal Reactivity Index; JSE—Jefferson Scale of Empathy; JSI—Job Satisfaction Indicators; JSPE—Jefferson Scale of Physician Empathy; JSE-S—the Jefferson Scale of Empathy—medical student version; M—male; MBTI—The Myers–Briggs Type Indicator; MIPS—Millon Index of Personality Styles; MOJAC—to measure moral orientation; MOS-SSS—MOS-Social Support Scale; MSCEIT ^TM^—Mayer–Salovey–Caruso Emotional Intelligence Test; NACE scale measures: Narcissism (N), Aloofness (A), (Self-)Confidence (C), and Empathy (E); NEO-FFI—the NEO-FFI Big Five personality model (neuroticism, extraversion, openness to experience, agreeableness and conscientiousness); NEO-PI—NEO Personality Inventory; NEO-PI-R—The NEO personality inventory revised; OCEAN personality: Openness, Conscientiousness, Extraversion, Agreeableness, and Neuroticism; PBL—problem-based learning; PQA—Personal Qualities Assessment; RAS—Rathus assertiveness schedule; RIPLS—modified Readiness of health care students for Interprofessional Learning Scale; RMET—Reading the Mind in the Eyes Test; SF—Trait Emotional Intelligence Questionnaire-Short Form; SIB—Scale for Interpersonal Behavior; SII—Strong Interest Inventory; SITIQ—Self-Implicit Theories of Intelligence Questionnaire; SWLS—Satisfaction With Life Scale; TEQ—Toronto Empathy Questionnaire; TEIQue-TMMS—Trait Meta–Mood Scale; UDO—universal diverse orientation; USMaP-i—USM Personality Inventory; WAIS—Wechsler adult intelligence scale; ZKPQ—Zuckerman-Kuhlman Personality Questionnaire; ZKPQ-S—Zuckerman–Kuhlman personality questionnaire—short form.
